# Custom target-sequencing in triple-negative and luminal breast cancer from young Brazilian patients

**DOI:** 10.1016/j.clinsp.2024.100479

**Published:** 2024-08-28

**Authors:** Pedro Adolpho de Menezes Pacheco Serio, Daniela Marques Saccaro, Ana Carolina Ribeiro Chaves de Gouvêa, Giselly Encinas, Simone Maistro, Gláucia Fernanda de Lima Pereira, Vinícius Marques Rocha, Larissa Dias de Souza, Viviane Jennifer da Silva, Maria Lucia Hirata Katayama, Maria Aparecida Azevedo Koike Folgueira

**Affiliations:** a Comprehensive Center for Precision Oncology (C2PO), Centro de Investigação Translacional em Oncologia (CTO), Departamento de Radiologia e Oncologia, Instituto do Câncer do Estado de São Paulo, Hospital das Clínicas, Faculdade de Medicina, Universidade de São Paulo (HCFMUSP), São Paulo, SP, Brazil; b Centro de Oncologia e Doenças Autoimune (COE), São Jose dos Campos, SP, Brazil; c Agilent Brazil (Agilent Technologies), Alphaville Industrial, Barueri, SP, Brazil

**Keywords:** Young Adults, Breast Cancer, Driver Genes

## Abstract

**Objectives:**

To identify somatic mutations in tumors from young women with triple-negative or luminal breast cancer, through targeted sequencing and to explore the cancer driver potential of these gene variants.

**Methods:**

A customized gene panel was assembled based on data from previous sequencing studies of breast cancer from young women. Triple-negative and luminal tumors and paired blood samples from young breast cancer patients were sequenced, and identified gene variants were searched for their driver potential, in databases and literature. Additionally, the authors performed an exploratory analysis using large, curated databases to evaluate the frequency of somatic mutations in this gene panel in tumors stratified by age groups (every 10 years).

**Results:**

A total of 28 young women had their tumoral tissue and blood samples sequenced. Using a customized panel of 64 genes, the authors could detect cancer drivers in 11/12 (91.7 %) TNBC samples and 11/16 (68.7 %) luminal samples. Among TNBC patients, the most frequent cancer driver was TP53, followed by NF1, NOTCH1 and PTPN13. In luminal samples, PIK3CA and GATA3 were the main cancer drivers, and other drivers were GRHL2 and SMURF2. CACNA1E was involved in both TN and luminal BC. The exploratory analysis also indicated a role for SMURF2 in luminal BC development in young patients.

**Conclusions:**

The data further indicates that some cancer drivers are more common in a specific breast cancer subtype from young patients, such as TP53 in TNBC and PIK3CA and GATA3 in luminal samples. These results also provide additional evidence that some genes not considered classical cancer-causing genes, such as CACNA1E, GRHL2 and SMURF2 might be cancer drivers in this age group.

## Highlights

• In breast cancer from young women:
  TP53 was affected in 75 % of TN samples, in concomitance with at least one additional driver gene, mainly NF1, NOTCH1 or PTPN13.
• In TN tumors carrying a wild type TP53, other drivers were TSG, like ATR and NF1.
• PIK3CA and GATA3 were the main cancer driver genes in luminal samples, and candidate drivers were GRHL2 and SMURF2.
• CACNA1E is a candidate cancer driver in both luminal and TN samples.

## Introduction

Breast cancer is predominantly diagnosed in women with advanced age. Despite that, breast cancer is the most common cancer in young women aged 30 to 39 years and the most lethal cancer type in this age range.^1^

Young patients may have a worse prognosis compared with older patients. Very young women (≤ 35 years), diagnosed with luminal B, HER2 and triple-negative breast cancer subtypes were shown to have a reduced overall survival and/or disease-free survival than their older counterparts.^2^ Similar results were reported in a large cohort of 1,732 triple-negative patients, where the median overall survival was 7 years for younger patients and 12 to 14 years for older age groups.^3^

In Brazil, there are indications that breast cancer in young women is a serious problem. A study evaluating hospital registries from 188,753 Brazilian breast cancer patients against 922,962 breast cancer patients listed in the National Cancer Institute (SEER), suggested a higher prevalence of breast cancer in young patients in Brazil (Odds Ratio: 2.2). In addition, an analysis of Brazilian breast cancer mortality rates between 1996 and 2017, which included 19,105 young patients, reported an increase in breast cancer mortality in young adults for most Brazilian regions.^4^^,^^5^

There are indications that tumor characteristics in young women may present some distinctive features. It was shown that those patients may present enriched gene expression signatures related to stem cells, growth factors, and the immune system.^3^ More recent studies highlight the importance of the immune system in young women, suggesting that such patients may have a more active Tumor Microenvironment (TME).^6^ Following these findings, some studies have also reported specific TME signatures in young adults with breast cancer.^7^^,^^8^

There are reports that the TNBC subtype may present distinctive features in young adult patients.^8^^,^^9^ For example, gene expression profiling suggests that the LAR (Luminal Androgen Receptor) subtype is less frequent in younger patients, showing that age may directly or indirectly influence the molecular profile.^6^

The studied group recently explored the mutational profile of both TN and luminal breast cancer subtypes in young adults. In TNBC, the median number of genes listed as oncogenes and/or tumor suppressor genes in the Cancer Gene Census (CGC) was three, in younger patients, while it was twice this value, in elderly patients. The majority (72 %) of TNBC samples presented at least one affected oncogene in association with at least one affected tumor suppressor gene. In TNBC samples from young women, the gene most frequently affected was TP53, detected in 70 % of the tumors, followed by other oncogenes and tumor suppressor genes, such as PIK3CA, KMT2C, and NF1. In addition, 20 % of tumors had mutations in genes involved in the RAS or PIK3CA signaling pathways. Some potential tumor suppressor genes with likely loss-of-function variants were also described, such as PHF6, GRIN2A, PIK3R1, and MED12. Furthermore, these tumors showed a predominance of the Mutational Signature 3, which is related to DNA Homologous Repair Defects (HRD).^10^ Other studies have also demonstrated a frequent co-occurrence of pathogenic somatic variants in TP53 and mutational signatures in the HRD pathway in young patients.^11^

The authors have also evaluated the molecular characteristics of luminal BC in a cohort of Brazilian young patients, in addition to data available in databases, from studies previously performed. In luminal BC from young women, the median mutation rate was 1.9 Mbp per sample and the most frequent event was C to T base transitions. The most frequently affected known cancer driver genes were PIK3CA, TP53, PRKAR1A, POLD1 and GATA3. Besides that, some affected new potential cancer drivers (genes that were not cataloged at CGC) were identified, such as CACNA1E, GRHL2, MTHFD2, PIK3AP1, RSBN1, SEMA6D, and SMURF2. There was a predominance of mutational Signature 1 (age-related).^12^

In the present study, the main goal was to further evaluate somatic gene variants in TN and luminal breast cancer samples from young Brazilian patients, through targeted sequencing and to explore the driver potential of these variants.

## Patients and methods

### Patients

Patients were prospectively included at Instituto do Câncer do Estado de São Paulo (ICESP), São Paulo, Brazil, from 2016 to 2021. This study was approved by the Research Ethics Committee of the Faculdade de Medicina da Universidade de São Paulo and followed in accordance with the principles of the Helsinki Declaration (CAAE: 54689316.9.0000.0065 and CAAE: 88763218.0.0000.0065).

Inclusion criteria were young female patients diagnosed with Triple-Negative (TN) or luminal (HER2 negative) breast cancer, aged 18 to 40 years at diagnosis, without previous cancer treatment, who had Formalin-Fixed Paraffin-Embedded (FFPE) tumor samples collected during biopsy or breast surgery available for the study. Patients who agreed to participate in the study and met the inclusion criteria signed the informed consent, and provided a peripheral blood sample.

### Customized gene panel

The authors assembled a customized gene panel. The genes chosen comprised: 1) Genes previously described as frequently altered in breast cancer samples from young adults; 2) Oncogenes and tumor suppressor genes frequently affected in breast cancer. These genes were selected based on: 1) The Catalog of Somatic Mutations in Cancer (COSMIC), which is a specialized database; 2) Breast cancer literature indicating their involvement in cancer.^10^^,^^12^

In summary, we:

I. Selected studies in which breast cancer samples from young patients were analyzed through high throughput technologies (Whole Exome or Whole Genome Sequencing; WES or WGS).
II. Selected affected genes that: 1) Were cataloged in the Cancer Gene Census databases, as oncogene and/or tumor suppressor genes and/or; 2) Had literature showing their involvement in cancer and/or; 3) Showed truncated (frameshift, stop gain, canonical splice site) variants, in case of tumor suppressor genes.
III. Filtered the previously selected genes by their specific breast cancer frequency in COSMIC, as described in the “Calculate Mutation Frequencies” in https://cancer.sanger.ac.uk/cosmic/help/faq to select those with frequency >1 %.
IV. Excluded genes that were frequently mutated and: a) Encoded large-sized proteins and/or b) Showed a high number of paralogs; factors that might introduce bias in the frequency of mutation, according to a previous report.^13^

The final panel comprised a total of 6928 probes and a size of 489 kbp, representing all coding regions of 64 genes, including 10 bases of non-coding sequences at the 5′ and 3′ ends of each exon. The sequences were designed based on the human genome reference GRCh37. Table 1 shows the gene panel, classified as Tumor Suppressor Gene (TSG), Oncogene (OG), and dual role (TSG or OG), according to the Cancer Gene Census database, as well genes not yet classified in CGC, but previously shown to be mutated in breast cancer.

**Table 1:** . Genes integrating the personalized gene-panel, classified according to the Cancer Gene Census.

Non_CGC	TSG	OG	Dual
AHNAK	APC	AKT1	ESR1
ANLN	ARID1A	ERBB2	GATA3
ATAD2B	ATM	ERBB4	MAP2K4
ATXN1	ATR	GRM3	MAP3K1
CACNA1E	BAP1	MET	NOTCH1
CAMK1G	BRCA1	MTOR	PRKAR1A
CSPP1	BRCA2	PIK3CA	TP53
DALRD3	CDH1	TNC	‒
FAT2	CSMD3	UBR5	‒
GRHL2	DICER1	‒	‒
HERC2	EP300	‒	‒
HUWE1	FAT4	‒	‒
LYST	FBXW7	‒	‒
MED23	MEN1	‒	‒
MTHFD2	NF1	‒	‒
NCOA3	PIK3R1	‒	‒
PARP4	POLD1	‒	‒
PCDH10	PTEN	‒	‒
PIK3AP1	PTPN13	‒	‒
PRKD1	RB1	‒	‒
RAD51	SETD2	‒	‒
RAD9A	SMARCA4	‒	‒
RSBN1	SPEN	‒	‒
SEMA6D	‒	‒	‒
SMURF2	‒	‒	‒

In this gene panel, seven genes are known clinically actionable genes, listed in the “National Cancer Comprehensive Network (NCCN) guidelines for Genetic/Familial High-Risk Assessment in breast-ovarian and pancreatic cancer (v 2.2022)”, i.e.: ATM, BRCA1, BRCA2, CDH1, NF1, PTEN, and TP53. Besides that, 40 out of the total 64 genes (62.5 %) are considered cancer genes by OncoKB™.

### DNA extraction and sequencing

Patients who agreed to participate in the study had their respective DNA extracted from Formalin-Fixed Paraffin-Embedded (FFPE) tumor and peripheral blood samples using the QIAamp DNA FFPE Tissue (Qiagen ‒ 56404) and QIAamp DNA Mini Kit (Qiagen – 51306), respectively, following the manufacturer's protocol.

Library preparation was conducted as described in the SureSelectXT HS Target Enrichment System for Illumina Multiplexed Sequencing Platforms protocol (Agilent) and sequenced on the NextSeq device (NextSeq 500/550 Mid Output Kit v2.5, 150 cycles; Illumina).

The median coverage of target areas was higher than 330 reads for all blood samples and 167 reads and 78 reads for TN and luminal tumor samples, respectively.

### Data processing after sequencing

The authors used the SureCall software (v.4.2.2; Agilent) with its base settings to perform a) Quality trimming (to remove low-quality bases and adapters; b) Alignment with the reference genome GRCh37 (BWA MEM); c) Removal of duplicates; d) Somatic CNV (Copy Number Variation) and variant call analysis.

Somatic and germline variant classification was performed with the SureCall Duo Analysis. Summarily, variants that passed quality control (Supplementary Methods) and were identified in both paired samples (FFPE tumor and blood) were classified as germline, while variants exclusively identified in the tumor sample were classified as somatic.

### Classification of variants

a) Germline variants: Germline variants were classified as benign, likely-benign, Uncertain Significance (VUS), likely-pathogenic, or pathogenic, following the American College of Medical Genetics and Genomics (ACMG) classification.^14^ BRCA1 variant effects were also verified in the study published by Findlay and colleagues.^15^ b) Somatic variants:

1b) Somatic variants were classified as causing Likely Loss-of-Function (L-LOF), Loss-Of-Function (LOF), Likely Gain-of-Function (L-GOF) or Gain-Of-function (GOF), in accordance with three curated databases: OncoKB (https://www.oncokb.org/; Accessed January 2022); TP53 Database (https://tp53.isb-cgc.org/; Accessed January 2022) and CancerVar tool (https://cancervar.wglab.org/; Accessed January 2022).^16^

2b) Gene variants not classified in OncoKB and TP53 Databases were considered as Probably Pathogenic (PP), if it was a:

I – Truncated variant (frameshift, nonsense, and canonical splice-site) in any gene.

II ‒ Missense variant that followed the criteria below:

1) The gene was cataloged as a CGC gene and the gene variant was predicted to be damaging in at least 2 out of 8 prediction tools and 1 out of 4 integration tools, or;

2) The gene was not cataloged as a CGC gene, but the gene variant was predicted to be damaging in at least 4 out of 8 prediction tools and 2 out of 4 integration tools (Supplementary Methods). This evaluation used a) *In-silico* variant effect prediction tools: FATHMM, MutationAssesor, MutationTaster, PROVEAN, Polyphen2 HDIV, Polyphen2 HVAR, SIFT and SIFT4G, and b) Variant effect integration tools: REVEL, METALR, METASVM, and MCAP, which are algorithms that focus on the integration of results from multiple variant effect prediction tools, and represent a more robust variant classification.

Loss of Heterozygosity (LOH) in BRCA1 and BRCA2 was analyzed as proposed by Jonge and colleagues.^17^ Briefly, BRCA1 and BRCA2 Variant Allele Frequency (VAF) were compared between tumor and normal samples (peripheral blood) pairs. LOH was considered true if a tumor with at least 20 %> malignant cellularity had a BRCA1/2 variant with a VAF > 60 %.

### In-silico analysis of tumor data from BC patients in different age groups

The authors analyzed high throughput sequencing data already available in COSMIC and cBioPortal databases. For this analysis, breast cancer samples from patients of all ages were divided into five age groups for comparison (≤ 40, considered young adults; 41‒50; 51‒60; 61‒70 and > 70).

The Cancer Browser Tool from COSMIC (https://cancer.sanger.ac.uk/cosmic/browse/tissue; v.96; Accessed May 2022) and the cohort comparison tools from the cBioPortal (https://www.cbioportal.org/; Accessed June 2022) were used to perform exploratory analyses, integrating data from multiple breast cancer exome and genome sequencing studies. Following the instructions for mutant frequency calculation with data from COSMIC (available at: https://cancer.sanger.ac.uk/cosmic/help/faq), the authors downloaded the GRCh37 version of the breast cancer Targeted and Genome Screens and Samples data files from the portal (https://cancer.sanger.ac.uk/cosmic/download; Accessed: May 2022) and processed in R (v. 4.1.2).

In the cBioPortal database, the analysis analysis was focused on samples from the TCGA Breast Cancer Study, (GRCh37, https://www.cbioportal.org/study/summary?id=brca_tcga) and conducted with the portal built-in cohort comparison tools. The p-values obtained from comparisons of samples in the COSMIC analysis (Fisher's Exact Test) were adjusted (adj.p) with the Bonferroni correction, and in the cBioPortal built-in analysis (Kruskal-Wallis, Chi-Squared and Student's *t-*test) were corrected with the Benjamini-Rochberg procedure.

The authors also evaluated gene expression and protein expression in different age groups. However, this analysis was exclusively done in the TCGA breast cancer cohort because data on CNV, gene expression and protein expression were only available in this databank.

Additional quality control, data processing, filtering and complementary annotations are described in the Supplementary Methods.

## Results

### Characteristics of the patients

Paired blood and tumor samples from 28 young BC patients were analyzed, including 12 TNBC, 13 luminal B and 3 luminal A. Patients had a median age at diagnosis of 33 years and all of them were diagnosed with invasive ductal carcinoma. Most tumors were histological grades 2 (61 %) and 3 (35 %). Among the patients, 71 % (20/28) reported a Family History (FH) of any cancer (until third-degree relatives), including 32 % (9/28) who had a positive FH of at least one relative (until third-degree) with the diagnosis of breast, ovary, pancreas and/or prostate cancer (Table 2).

**Table 2:** Clinical data summary.

	**Patients (n** = **28)**		
	**TNBC (n** = **12)**	**Luminal (n** = **16)**	**p-value**
**Age (median)**	36	33	NS
**Tumor Grade, n (%)**			
I	0 (0 %)	1 (6 %)	NS
II	5 (42 %)	12 (75 %)	
III	7 (58 %)	3 (19 %)	
**Family History, n (%)**	5 (42 %)	4 (25 %)	NS
**BRCA1/2 Status, n (%)**			
LP/P	1 (8 %)	3 (19 %)	NS
VUS	1 (8 %)	0 (10 %)	

LP, Likely-Pathogenic; P, Pathogenic; VUS, Variant of Uncertain Significance. The variants are classified following the ACMG germline classification criteria and exported from Clinvar. Family history, Family history of at least one relative (until third-degree relatives) with a diagnosis of breast, ovary, pancreas, or prostate cancer.

### Germline variants

Four out of the 28 (14 %) patients were germline P/LP variant carriers in BRCA1 or BRCA2. Among the 12 TNBC patients, one presented a P/LP variant in BRCA1 (c.245T>G) (Table 3). Besides that, half of the TNBC patients presented one or more VUS, as shown in Supplementary Table 1. In total, 61 germline variants were identified among TNBC patients.

**Table 3:** Pathogenic and likely-pathogenic germline variants.

**ID**	**Cancer_type**	**Gene**	**Variant_type**	**AA_change**	**cDNA**	**VAF_germ**	**VAF_som**	**ACMG**	**ACMG_criteria**
635	TNBC	BRCA1	Missense	p.L82R	c.245T>G	0.427	0.738	LP	PS3; PM2; PP3
811	LUMINAL	BRCA1	Splice_donor	NA	c.441+2T>A	0.528	0.531	P	PP5; PVS1; PM2; PP3
821	LUMINAL	BRCA1	Frameshift_ins	p.G1777fs	c.5266dupC	0.505	0.592	P	PP5; PS3; PVS1; PM2
842	LUMINAL	BRCA2	Nonsense	p.Q84*	c.250C>T	0.473	0.516	P	PVS1; PS3; PM2

AA_change, Amino Acid Change; VAF_germ, Germline Variant allele Frequency; VAF_som, Somatic Variant allele Frequency; ACMG, Germline Variant Classification (American College of Medical Genetics and Genomics guidelines); P, Pathogenic; LP, Likely-Pathogenic.

Among the 16 luminal BC patients, there were two BRCA1 P/LP variant carriers: one frameshift, c.5266dup; p.Q1777fs, and one splice-site variant c.441+2T>A;, plus one BRCA2 P/LP variant carrier, a stop-codon gain (c.250C>T) (Table 3). None of these three patients reported a family history of cancer. Another six patients were VUS carriers in various genes (Supp. Table 1; Supp. Fig. 1). In total, 77 germline variants were identified among luminal patients.

### Somatic variants: general view

The median number of somatic variants per sample was 2.5 for TNBC and 2 for luminal BC. In one TNBC sample and 4 luminal samples neither somatic single nucleotide variants, nor indels or CNVs were detected. One luminal tumor presented only a somatic CNV.

**Fig. 1:**
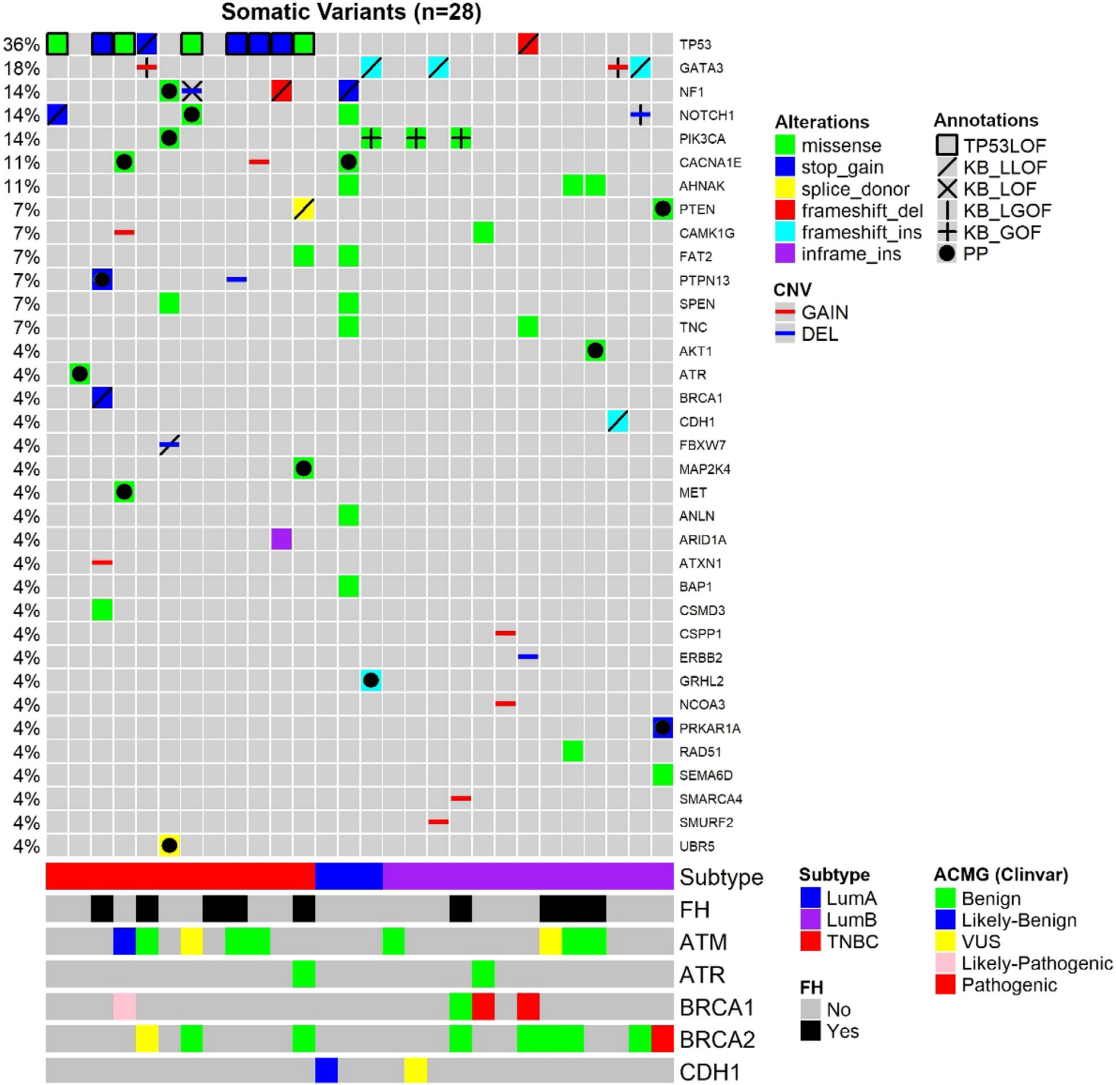
**Oncoplot of the somatic variants detected in the 28 young adult breast cancer patients.** Each column is a patient, and each line represents a gene. Annotations: TP53 variants classified as causing LOF (TP53 Database), variants classified according to curated studies in OncoKB (KB_LLOF, KB_LOF, KB_LGOF, KB_GOF); PP, Probably Pathogenic variant, according to variant type (truncating variants) or variant effect prediction tools (missense variants); FH, Family History of at least one relative (until third-degree relatives) with a diagnosis of breast, ovary, pancreas, or prostate cancer; ACMG, Classification of germline variants. For patients with two variants in the same gene, the variant with the higher effect (driver or probably driver) was plotted.

Considering both TN and luminal samples, somatic variants were detected in a total of 35 different genes (Table 4). Among all samples, the most frequently affected gene was TP53, altered in 10 out of 28 tumor samples (36 %), followed by GATA3 in 5 samples (18 %) and PIK3CA, in 4 samples (14 %) (Fig. 1; Table 4, Table 5).

**Table 4:** Somatic variants from young adult breast cancer patients.

**Subtype**	**ID**	**Gene**	**AA/chr_arm**	**Consequence**	**rs**	**ONCOKB**	**Cancer_var**	**TP53_DB**	**PRED**	**COMP_PRED**	**CGC**	**Int**
TNBC	605	**NOTCH1**	Q2393*	**Stop_gained**	rs779613930	**LLOF**	NA	NA	NA	NA	OG/TSG/Fus	**P**
TNBC	605	**TP53**	S241P	Missense	rs1057520002	NA	**TIER_1_STRONG**	**LOF**	8	4	OG/TSG/Fus	**P**
TNBC	608	**ATR**	D455Y	Missense	‒	NA	TIER_3_UNCERTAIN	NA	**6**	**1**	TSG	**PP**
TNBC	611	**ATXN1**	chr6:p22.3	Cnv_gain	‒	‒	‒	‒	‒	‒	-	**-**
TNBC	611	**BRCA1**	C1718-1719*	**Stop_gained**	rs80357710	**LLOF**	NA	NA	NA	NA	TSG	**P**
TNBC	611	CSMD3	D3476H	Missense	-	NA	TIER_4_BENIGN	NA	2	0	TSG	**-**
TNBC	611	**PTPN13**	S839*	**Stop_gained**	-	NA	NA	NA	NA	NA	TSG	**PP**
TNBC	611	**TP53**	W91*	**Stop_gained**	rs876660548	**LLOF**	NA	**LOF**	NA	NA	OG/TSG/Fus	**P**
TNBC	635	CACNA1E	A2109S	Missense	rs201622587	NA	ND	NA	2	3	NA	**-**
TNBC	635	CAMK1G	chr1:q32.2	Cnv_gain	‒	‒	‒	‒	‒	‒	-	-
TNBC	635	MET	P356T	Missense	‒	NA	TIER_3_UNCERTAIN	NA	6	0	OG	**-**
TNBC	635	**MET**	T582I	Missense	‒	NA	TIER_3_UNCERTAIN	NA	**7**	**4**	OG	**PP**
TNBC	635	**TP53**	R248W	Missense	rs121912651	**LOF**	**TIER_1_STRONG**	**LOF**	8	4	OG/TSG/Fus	**P**
TNBC	700	**GATA3**	chr10:p14	Cnv_gain	‒	**LGOF**	‒	‒	‒	‒	-	**P**
TNBC	700	**TP53**	C141*	**Stop_gained**	rs1057519977	**LLOF**	NA	not_LOF	NA	NA	OG/TSG/Fus	**P**
TNBC	701	**FBXW7**	chr4:q31.3	Cnv_loss	‒	**LLOF**	‒	‒	‒	‒	-	**P**
TNBC	701	**NF1**	P1087T	Missense	‒	NA	TIER_3_UNCERTAIN	NA	**7**	**1**	TSG/Fus	**PP**
TNBC	701	**PIK3CA**	E707K	Missense	rs3729687	NA	**TIER_2_POTENTIAL**	NA	**7**	**4**	OG	**PP**
TNBC	701	SPEN	H2391Y	Missense	‒	NA	TIER_4_BENIGN	NA	1	0	TSG	**-**
TNBC	701	**UBR5**	-	**Splice_donor**	‒	NA	NA	NA	NA	NA	OG	**PP**
TNBC	702	**NF1**	chr17:q11.2	Cnv_loss	‒	**LOF**	‒	‒	‒	‒	‒	**P**
TNBC	702	**NOTCH1**	R2087W	Missense	rs373806373	NA	**TIER_2_POTENTIAL**	NA	**7**	**2**	OG/TSG/Fus	**PP**
TNBC	702	**TP53**	V216E	Missense	rs1057520004	NA	**TIER_1_STRONG**	**LOF**	8	4	OG/TSG/Fus	**P**
TNBC	715	**PTPN13**	chr4:q21.3	Cnv_loss	‒	‒	‒	‒	‒	‒	‒	‒
TNBC	715	**TP53**	Q317*	**Stop_gained**	rs764735889	**LLOF**	NA	**LOF**	NA	NA	OG/TSG/Fus	**P**
TNBC	719	**CACNA1E**	chr1:q25.3	Cnv_gain	‒	‒	‒	‒	‒	‒	‒	‒
TNBC	719	**TP53**	R306*	**Stop_gained**	rs121913344	**LLOF**	NA	**LOF**	NA	NA	OG/TSG/Fus	**P**
TNBC	728	ARID1A	E38A	Inframe_insertion	rs1266385064	NA	‒	‒	‒	‒	TSG	unknown
TNBC	728	**NF1**	S340A	Missense	‒	NA	TIER_3_UNCERTAIN	NA	**2**	**1**	TSG/Fus	‒
TNBC	728	**NF1**	A706V	Missense	‒	NA	TIER_3_UNCERTAIN	NA	**6**	**1**	TSG/Fus	**PP**
TNBC	728	**NF1**	L2345-2346X	Frameshift	‒	**LLOF**	NA	NA	NA	NA	TSG/Fus	**P**
TNBC	728	**TP53**	R342*	**Stop_gained**	rs1321845532	**LLOF**	NA	**LOF**	NA	NA	OG/TSG/Fus	**P**
TNBC	730	FAT2	A2587V	Missense	rs148551207	NA	TIER_4_BENIGN	NA	6	1	NA	
TNBC	730	**MAP2K4**	H227Y	Missense	-	NA	TIER_3_UNCERTAIN	NA	**7**	**4**	OG/TSG	**PP**
TNBC	730	**PTEN**	-	**Splice_donor**	rs1114167622	**LLOF**	NA	NA	NA	NA	TSG	**P**
TNBC	730	**TP53**	C275Y	Missense	rs863224451	**LLOF**	**TIER_1_STRONG**	**LOF**	8	4	OG/TSG/Fus	**P**
LUM	732	**PIK3CA**	H1047R	Missense	rs121913279	**GOF**	**TIER_2_POTENTIAL**	NA	3	1	OG	**P**
LUM	800	**GATA3**	S408X	**Frameshift**	rs752977342	**LLOF**	NA	NA	NA	NA	OG/TSG	**P**
LUM	800	SMURF2	chr17.q23.3	Cnv_gain	‒	‒	‒	‒	‒	‒	‒	‒
LUM	809	**PIK3CA**	E542K	Missense	rs121913273	**GOF**	**TIER_2_POTENTIAL**	NA	4	1	OG	**P**
LUM	809	SMARCA4	chr19.p13.2	Cnv_gain	‒	‒	‒	‒	‒	‒	‒	‒
LUM	811	CAMK1G	R466P	Missense	rs769916493	NA	NA	NA	1	0	NA	-
LUM	818	CSPP1	chr8.q13.2	Cnv_gain	‒	‒	‒	‒	‒	‒	‒	‒
LUM	818	NCOA3	chr20.q13.12	Cnv_gain	‒	‒	‒	‒	‒	‒	‒	‒
LUM	819	AHNAK	D1342A	Missense	-	NA	TIER_4_BENIGN	NA	7	1	NA	‒
LUM	819	AHNAK	D1342Y	Missense	rs776554777	NA	TIER_3_UNCERTAIN	NA	7	1	NA	‒
LUM	819	ANLN	A40V	Missense	‒	NA	NA	NA	0	0	NA	‒
LUM	819	BAP1	P598S	Missense	‒	NA	TIER_3_UNCERTAIN	NA	0	0	TSG	‒
LUM	819	**CACNA1E**	L1792M	Missense	‒	NA	NA	NA	6	4	NA	**PP**
LUM	819	FAT2	G4294S	Missense	rs1482787673	NA	TIER_4_BENIGN	NA	5	1	NA	-
LUM	819	NF1	E1181*	**Stop_gained**	‒	**LLOF**	NA	NA	NA	NA	TSG	**P**
LUM	819	NOTCH1	R1523H	Missense	rs367589813	NA	TIER_3_UNCERTAIN	NA	0	1	OG/TSG/Fus	‒
LUM	819	SPEN	T1756I	Missense	‒	NA	TIER_4_BENIGN	NA	1	0	TSG	‒
LUM	819	TNC	R1741Q	Missense	rs149299073	NA	TIER_3_UNCERTAIN	NA	4	0	OG	‒
LUM	821	ERBB2	chr17.q12	Cnv_loss	‒	‒	‒	‒	‒	‒	‒	‒
LUM	821	TNC	R1675M	Missense	‒	NA	TIER_4_BENIGN	NA	1	0	OG	-
LUM	821	**TP53**	P80X	**Frameshift**	‒	**LLOF**	NA	NA	NA	NA	OG/TSG/Fus	**P**
LUM	822	**GATA3**	H435X	**Frameshift**	‒	**LLOF**	NA	NA	NA	NA	OG/TSG	**P**
LUM	822	**GRHL2**	-76-77X	**Frameshift**	‒	NA	NA	NA	NA	NA	NA	**PP**
LUM	822	NF1	chr17.q11.2	Cnv_loss	‒	‒	‒	‒	‒	‒	‒	‒
LUM	822	**PIK3CA**	H1047R	Missense	‒	**GOF**	**TIER_2_POTENTIAL**	NA	**3**	**1**	OG	**P**
LUM	828	AHNAK	V3972I	Missense	‒	NA	TIER_4_BENIGN	NA	0	0	NA	‒
LUM	828	RAD51	V112A	Missense	rs777467455	NA	TIER_4_BENIGN	NA	1	0	NA	‒
LUM	829	AHNAK	N3135K	Missense	‒	NA	TIER_4_BENIGN	NA	0	0	NA	‒
LUM	829	**AKT1**	P423L	Missense	‒	NA	TIER_3_UNCERTAIN	NA	**7**	**4**	OG	**PP**
LUM	837	**CDH1**	L769X	**Frameshift**	rs766222121	**LLOF**	NA	NA	NA	NA	TSG	**P**
LUM	837	**GATA3**	chr10.p14	Cnv_gain	‒	**LGOF**	‒	‒	‒	‒	‒	**P**
LUM	838	**GATA3**	M443X	**Frameshift**	‒	**LLOF**	NA	NA	NA	NA	OG/TSG	**P**
LUM	838	**NOTCH1**	chr9.q34.3	Cnv_loss	‒	**LGOF**	‒	‒	‒	‒	‒	**P**
LUM	842	**PRKAR1A**	R96*	**Stop_gained**	rs281864783	NA	NA	NA	NA	NA	OG/TSG/Fus	**PP**
LUM	842	**PTEN**	L220V	Missense	‒	NA	TIER_3_UNCERTAIN	NA	**4**	**2**	TSG	**PP**
LUM	842	SEMA6D	I406T	Missense	‒	NA	NA	NA	6	0	NA	‒

ID, Sample ID; ONCOKB, Variant is reported at OncoKB as Likely-Loss-of-Function (L-LOF), Loss-Of-Function (LOF), Likely Gain-Of-Function (L-GOF) or gain-of-function causing, or not reported in OncoKB (NA); Cancer_var, Classification of somatic variants based on the tool developed by Li et. al.(2022); TP53_DB, TP53 somatic variants classification based on data from functional studies reported at TP53 Database; PRED, Number of variant effect prediction tools (max: 8) in which the variant was classified as damaging; COMP_PRED, Number of variant effect prediction integration tools (max: 4) in which the variant was classified as damaging; CGC, Cancer Gene Census role in Cancer; OG, Oncogene; TSG, Tumor Suppressor Gene; Fus, Fusion; NA, Not Available; INT, Interpretation; P, Pathogenic; PP, Probably pathogenic.

**Table 5:** Somatic variants detected in 28 young adult breast cancer patients, classified according to the Cancer Gene Census (CGC).

**ID**	**Subtype**	**Non_CGC**	**TSG**	**OG**	**Dual**
605	TNBC	-	-	-	TP53–, NOTCH1-
608	TNBC	-	ATR	-	-
611	TNBC	ATXN1 (GAIN)	BRCA1-, CSMD3, **PTPN13**	-	TP53–
635	TNBC	CACNA1E, CAMK1G (GAIN)	-	MET (PP)	TP53–
700	TNBC	-	-	-	TP53-, GATA3+ (GAIN)
701	TNBC	-	FBXW7-(DEL), NF1, SPEN,	PIK3CA (PP), **UBR5**	-
702	TNBC	-	NF1–(DEL)	-	TP53–, NOTCH1 (PP)
703	TNBC	-	-	-	-
715	TNBC	-	PTPN13 (DEL)	-	TP53–
719	TNBC	CACNA1E (GAIN)	-	-	TP53–
728	TNBC	-	ARID1A, NF1-	-	TP53–
730	TNBC	FAT2	PTEN-	-	TP53–, MAP2K4 (PP)
731	LumB	-	-	-	-
732	LumB	-	-	PIK3CA++	-
800	LumB	SMURF2 (GAIN)	-	-	GATA3-
809	LumB	-	SMARCA4 (GAIN)	PIK3CA++	-
811	LumB	CAMK1G	-	-	-
812	LumA	-	-	-	-
818	LumB	CSPP1 (GAIN), NCOA3 (GAIN)	-	-	-
819	LumA	AHNAK, ANLN, CACNA1E (PP), FAT2	BAP1, NF1-, SPEN	TNC	NOTCH1
821	LumB	-	-	ERBB2 (DEL), TNC	TP53-
822	LumA	**GRHL2**	NF1–(DEL)	PIK3CA++	GATA3-
823	LumB	-	-	-	-
828	LumB	AHNAK, RAD51	-	-	-
829	LumB	AHNAK	-	AKT1 (PP)	-
837	LumB	-	CDH1-	-	GATA3+(GAIN)
838	LumB	-	-	-	NOTCH1+(DEL), GATA3-
842	LumB	SEMA6D	PTEN (PP)	-	**PRKAR1A**

TSG, Tumor Suppressor Gene; OG, Oncogene; Dual, Dual-role gene (TSG and OG); GAIN, CNV gain; DEL, CNV deletion; -, Likely loss-of-function; –, Loss-of-function; +, Likely gain-of-function; ++, Gain-of-function; PP, Probably Pathogenic missense variant according to variant effect prediction tools); genes in bold: probably pathogenic truncating (frameshift, nonsense and canonical splice-site) variants.

Among TNBC samples, 36 somatic variants were detected. TP53 was the most frequently affected gene, followed by NF1. For somatic CNVs, 58 % of TNBC patients presented a single gain or loss (Fig. 1; Table 4, Table 5). In one tumor sample, from a BRCA1 germline variant carrier (p.L82R), the BRCA1 VAF was 0.738, suggesting a loss of heterozygosity LOH event, or a clonal expansion.^17^

Among luminal tumor samples, a total of 36 somatic variants were identified. The most frequently affected genes were GATA3 (4/16) and PIK3CA (3/16). Regarding CNVs, 50 % of the samples presented copy gain or loss, most frequently a single CNV, excluding one case, that harbored two CNVs (Fig. 1; Table 4, Table 5).

### Driver genes

The authors next evaluated the meaning of every gene variant to characterize driver genes and potential driver genes. The authors assumed that driver genes were those cancer-causing genes, as reported in CGC, OncoKB, or CancerVar databases, affected by pathogenic variants. Potential driver genes were cancer-causing genes, as defined above, or other genes implicated in cancer, according to the literature, that harbored probably pathogenic variants, as described in methods. Afterward, the authors considered the driver and potential driver genes, all together, as “cancer drivers” to make a final analysis of the number of “cancer drivers” per tumor.

In TNBC, the authors identified at least one affected driver gene in ten (83.3 %) of tumor samples plus one potential driver in one sample (8.3 %). The latter tumor presented a single alteration, which was a probably pathogenic mutation in ATR, that represents a TSG. Only one tumor did not present any driver or potential driver genes.

In TNBC, the most frequent driver gene was TP53, affected by loss of function variants in 75 % of the samples (Fig. 1; Table 4). The second most frequent drivers were two TSG, NF1 and PTPN13, which were affected by the loss of function variants in two tumors, each gene. NF1 was also a potential driver in a third tumor that harbored a probably pathogenic NF1 variant (Fig. 1; Table 4, Table 5). Other driver genes that were affected by loss of function variants were TSGs, such as BRCA1, FBXW7, and PTEN.

Among oncogenes, missense probably pathogenic variants were detected in MET and PIK3CA, which were thus considered potential driver genes. In addition, a splice donor variant was detected in the oncogene UBR5 (CGC).

The authors next evaluated the number of “cancer drivers” in each tumor. Nine TNBC samples presented more than one somatic cancer driver, including five, that revealed two cancer drivers, represented by one dual-role gene (TP53) in concomitance with one TSG (NF1 or PTPN13), or one oncogene (MET), or one dual-role gene (NOTCHor1 or GATA3) (Table 4, Table 5). Another two samples presented three cancer drivers, consisting of TP53 in association with two dual-role genes (NF1 and NOTCH1) or with one dual role gene (MAP2K4) plus one TSG (PTEN). Another two samples presented four cancer drivers, consisting of TP53 in concomitance with three TSG (ATXN1, PTPN13, and BRCA1) in one of the tumors, and two TSGs (NF1 and FBXW7) in association with two oncogenes (PIK3CA and FBXW7) in the other tumor.

In luminal BC, 11 out of 16 samples presented cancer driver genes. The most frequent driver genes were in the dual role gene GATA3, mutated in four tumors, and oncogene PIK3CA, affected by the gain of function variants in three tumors (3/16) (OncoKB) (Fig. 1; Tables 4 and 5).

Two luminal samples presented loss of function variants in TSGs, NF1 or CDH1. Although two samples harbored variants in the dual role gene NOTCH1, only one variant was considered driver, i.e., a deletion associated with likely GOF (Fig. 1; Table 4, Table 5). Probably pathogenic missense variants were identified in the TSG PTEN, in the oncogene AKT1and in a gene other than CGC, CACNA1E (Fig. 1; Table 4, Table 5). Some other genes not yet listed in the CGC database that were affected were: GRHL2 (frameshift variant), SMURF2, CSPP1, and NCOA3 (all presenting copy number gain). In addition, the oncogene ERBB2, presented a copy number loss, and the TSG SMARCA4 presented a copy number gain.

Among three luminal A tumor samples, the number of “cancer drivers” varied from zero to three. One tumor presented three driver genes, consisting of GATA3 (dual role), PIK3CA (oncogene), and GRHL2 (gene other than CGC); another tumor, presented two driver genes represented by NF1 (TSG) and CACNA1E (gene other than CGC); and the third one, did not present any potential drivers among the sequenced gene panel.

Among 13 luminal B samples, two tumors presented just one cancer driver, i.e., PIK3CA (oncogene) in one of the tumors, and AKT1 (oncogene) in the other. Seven tumors presented two driver genes, including a) GATA3 (dual role gene) in three samples, associated with CDH1 (TSG), or NOTCH1 (dual role gene), or SMURF2 (gene other than CGC); b) TP53 (dual role) associated with ERBB2 (oncogene) in a BRCA1 germline pathogenic variant carrier; c) PRKAR1A (dual role) associated with PTEN (TSG), in a BRCA2 germline pathogenic variant carrier; d) PIK3CA (oncogene) associated with SMARCA4 (TSG, in this case, copy number gain); e) CSPP1 and NCOA3 (genes other than CGC, both presenting copy number gain). Four tumors did not present any somatic driver genes represented in this gene panel, despite that, one of these patients was a BRCA1 germline pathogenic variant carrier.

### Exploratory analysis

The authors used COSMIC and cBioPortal curated data to explore the relevance of alterations in the 64 genes evaluated in the present gene panel, in data deposited from other tumors. These databases were used to compile data from multiple breast cancer exome and genome sequencing projects. Patient tumor data was compared among 5 age groups (≤40, 41‒50, 51‒60, 61‒70 and >70) to compare mutation rates. The authors also investigated CNV, gene expression, protein expression, and methylation among age groups in the TCGA Breast Cancer Firehose Legacy Cohort (TCGA-BRCA).

At first, the authors explored whether there were differences in the frequency of point and truncated gene variants (missense, inframe, nonsense, frameshift, and splice-site) among age groups.

The authors accessed breast cancer data available at the COSMIC Mutation Data (Genome Screens) cohort (https://cancer.sanger.ac.uk/cosmic/download; Accessed: May 2022), comprising 2672 samples from multiple studies. Next, the authors excluded samples from case-report and cell-line studies, as well as samples of adenoid, acinic, neuroendocrine, metaplastic, and non-primary carcinoma, and samples with no information of age at diagnosis. The authors ended with data from 7 studies^12^^,^[18], [19], [20], [21], [22], [23] comprehending: ≤ 40y: n = 184; 41‒50y: n = 265; 51‒60y: n = 316; 61‒70y: n = 307; ≥ 71y: n = 225. No tumor subtype distinction was done since this kind of data was missing in some studies.

The authors then created a contingency table to compare the frequency of affected gene variants among age groups. The authors verified that CDH1 point and truncated variants were more frequently found in all elderly groups compared to young adults (≤ 40y) (adj.p < 0.001). Similarly, MAP3K1 presented higher mutation frequency in almost all the elderly groups (excluding 41‒50) in comparison to the young adults group (adj.p < 0.05).

A higher frequency of variants in TP53 (adj.p < 0.05) was identified in the elderly groups with less advanced ages (41‒50 and 51‒60) in comparison with the younger adults. Regarding PIK3CA, only the group with the most advanced age (> 70) presented higher variant frequency (adj.p = 0.003) in comparison to young adults.

The authors performed a similar analysis in breast cancer samples from the TCGA-BRCA cohort, divided into five age groups, as described, using the cohort comparison workflow from cBioPortal, comparing gene and protein expression, copy-number variation, and methylation profile between young adults and elderly patients. The cohort comprised 76 young adults and 992 elderly patients, divided as follows: 41‒50 (n = 230), 51‒60 (n = 273), 61‒70 (280) and > 70 (n = 209).^24^ No tumor subtype distinction was made since this data was missing in some tumors and further subgroup divisions would weaken the power of statistical analysis.

When comparing young adults with all the elderly groups (41‒50, 51‒60, 61‒70 and > 70), the young adult group presented a higher frequency of somatic copy-number gain in SMURF2 and PRKAR1A (adj.p < 0.05) with concomitant higher gene expression (adj.p<0.05), when compared to the most advanced age groups (61‒70 and > 70). Finally, the authors observed a higher (adj.p < 0.05) CDH1 gene and protein expression in young adults when compared to all the elderly subgroups. In addition, there was a higher gene expression in tumors from young adults when compared to the > 70y group for the following genes: ARID1A, ANLN, ATAD2B, FAT2, FAT4, FBXW7, MET, MTOR, PARP4, PIK3CA and RAD51.

## Discussion

In this cohort of young breast cancer patients, 14 % were BRCA1 or BRCA2 P/LP variant carriers, which stands in accordance with other reports, including one published by the group, showing a 16.5 % BRCA mutation prevalence among 79 luminal breast cancer young patients (below 36 years).^12^^,^^24^^,^^25^

The main goal was to identify somatic “cancer drivers” in TN and luminal BC samples from young patients, using targeted-sequencing analysis of a panel of genes. Among TNBC, the authors identified at least one cancer driver in 83 % of the tumor samples. The most frequently affected driver genes were TP53 and NF1, altered in 75 % and 25 % of the samples, respectively. Other driver genes were ATR, BRCA1, FBXW7, NF1, PTEN, PTPN13 (all six, TSG) and MET, PIK3CA and UBR5 (all three, OG). These findings are in accordance with those reported in previous studies on TNBC.^6^^,^^10^^,^^22^ All nine tumors with mutated TP53 also harbored a second cancer driver. Two TN tumors that carried a wild-type TP53, presented at least one affected oncogene, i.e., AKT1 or PIK3CA, but the third TP53 wild-type tumor did not present an identified driver through this panel of genes.

PTPN13 was exclusively affected in TNBC tumors (but not luminal samples), both TP53 mutated. PTPN13 encodes a protein tyrosine Phosphatase (PTP) and is classified as a tumor suppressor gene, with roles in apoptosis signaling regulation. There is data showing that PTPN13 dysfunction (LOF) in *in-vivo* and *in-vitro* TNBC models leads to enhanced malignant growth and invasiveness.^26^ The present TNBC tumor sample (611) presented a stop-codon variant, localized at the FERM-c domain. Although to our knowledge there is no study that investigated the functional impact of this specific variant, supportive data show that disruptive variants at the PTPN13 PDZ (protein-protein interaction) and protein tyrosine-phosphatase domains would disrupt its main antitumoral functions.^27^ Additionally, PTPN13 is frequently epigenetically inhibited in breast cancer.^28^ These facts highlight the relevance of this gene in breast cancer, although not necessarily exclusively in young adults, as it is not shown to be highly altered in the present study nor differentially expressed or methylated between age groups in the exploratory analysis.

One TNBC tumor had a stop-gain in NOTCH1, a dual-role gene (OG/TSG). This variant occurred in the C-terminal PEST domain, which in normal conditions leads to the Notch1 protein degradation, through the ubiquitin-proteasome pathway. Thus, mutations in the PEST domain may induce Notch oncogenic intracellular accumulation.^29^ In another TNBC, impairment of FBXW7, which encodes an E3 ubiquitin-ligase responsible for the ubiquitin-mediated NOTCH1 degradation, may also lead to oncogenic NOTCH1 accumulation.^30^ In this TN tumor, FBXW7 CNV loss occurred in concomitance with a splice-site variant in another E3 ubiquitin ligase gene, UBR5. Studies report that UBR5 overexpression may influence oncogenic pathways related to tumor growth, invasion and immune evasion in TNBC.^31^ Thus, the function of this alteration in tumor development is not clear.

CACNA1E was affected in two TNBCs, and one of these tumors presented a copy gain. CACNA1E encodes a calcium voltage-gated channel subunit alpha-1 E, which was hypothesized to act as an oncoprotein in non-small cell lung cancer, required for cell proliferation. CACNA1E copy gain was previously reported in gastrointestinal stromal tumors^32^ and Wilms' tumors.^33^ Besides that, CACNA1E mutation rate was associated with a patient's lifetime benzo(a)pyrene exposure, in lung cancers.^34^ CACNA1E mutation was also detected in luminal BC from young patients in the present and in a previous work of the studied group.^12^ All these data indicate that CACNA1E is probably a cancer driver in BC for young women.

Among luminal BC, 69 % of the samples presented somatic “cancer drivers”. The most frequently affected driver genes were GATA3 and PIK3CA, altered in 25 % and 18.7 % of the samples, respectively, in accordance with other studies in luminal BC in young women.^35^ PIK3CA missense variants leading to an oncogene gain of function is a classical feature of luminal breast cancer in both young and elderly patients. Despite that, in a previous study, the authors identified a trend toward an increased PIK3CA somatic mutation rate in ER-positive tumors in aging patients compared to younger patients.^18^^,^^36^

There is some data indicating that GATA3 alterations are more frequently observed in luminal BC from young patients than elderly patients.^35^ In the present series of luminal tumors, GATA3 was mainly inactivated through frameshift somatic variants.^12^^,^^18^ This pattern of GATA3 variants reproduces findings from studies that mainly included Caucasian patients but disagree with data from luminal samples from Asian patients, who mainly present GATA3 missense alterations.^18^^,^^35^^,^^37^ In luminal BC, there are also reports showing that, in general, GATA3 alterations are not associated with PIK3CA or TP53 alterations in the same tumor.^12^^,^^37^ Despite that, in the present study, among four samples presenting GATA3 alterations, one luminal A tumor also presented an activated PIK3CA.

Other “cancer drivers” in luminal BC from young patients were CDH1, NF1 and PTEN, which are classical TSG associated with breast cancer predisposition, as well as AKT, a classical BC oncogene.^18^

Among genes that are not listed as cancer genes in “Cancer gene census” it is worth mentioning GRHL2 and SMURF2.

GRHL2 is described in several breast cancer-related articles, mainly in luminal BC. Some authors report that GRHL2 exhibits important roles in hormone-dependent cancer, such as luminal breast cancer, affecting EMT processes and tumor progression.^38^

In the exploratory analysis using cancer databases the authors identified SMURF2 as more frequently amplified in tumors from young breast cancer patients when compared to elderly age groups (BRCA-TCGA). SMURF2 was previously identified as downregulated in triple-negative breast cancer.^39^ Moreover, a CNV gain in SMURF2 was detected in one luminal sample analyzed in the present study.

The present report presents some limitations. The authors suggest a missense variant classification based on variant effect prediction tools, for genes previously recognized as cancer genes in the CGC database or in the literature. The idea was to advance the recognition of potential drivers, however, it is not possible to clearly state whether the variant will cause a gain or loss of function (if any) in the protein, as well as how the result would be interpreted in an oncogene or in a TSG scenario. Similarly, CNVs findings were not further validated. Another limitation is the small sample size and limited gene panel. Despite that, most studies report data from patients with European ancestry. Thus, the strength of this study is to contribute additional somatic data on tumors from young patients from the Brazilian population, characterized by an admixed ancestry.^40^

## Conclusion

Sequencing a panel of genes, that included the most frequently altered genes in breast cancer, the authors identified at least one somatic cancer driver for 82 % of the tumors from young patients. The present data further indicates that some drivers are more common in a specific breast cancer subtype from young patients, such as TP53 in TNBC and PIK3CA and GATA3 in luminal samples. These results also provide additional evidence that genes such as PTPN13, in TNBC and SMURF2, GHRL2 and PRKAR1A, in luminal samples might be cancer drivers in this age group.

## Data availability

All data supporting the study findings are available within the paper's main and supplementary data.

## Authors' contributions

Pedro Adolpho de Menezes Pacheco Serio: Conceived the study, wrote the original draft, included patients, performed laboratorial and formal analysis and data curation, revised and approved the final manuscript.

Daniela Marques Saccaro: Conceived the study, included patients, performed laboratorial and formal analysis, revised and approved the final manuscript.

Ana Carolina Ribeiro Chaves de Gouvea: Included patients, revised and approved the final manuscript.

Giselly Encinas: Conceived the study, performed formal analysis, revised and approved the final manuscript.

Simone Maistro: Included patients, performed laboratorial analysis, revised and approved the final manuscript.

Gláucia Fernanda de Lima Pereira: Included patients, revised and approved the final manuscript.

Vinícius Marques Rocha: Performed laboratorial and formal analysis, revised and approved the final manuscript.

Larissa Dias de Souza: Performed laboratorial and formal analysis, revised and approved the final manuscript.

Viviane Jennifer da Silva: Performed laboratorial and formal analysis, revised and approved the final manuscript.

Maria Lucia Hirata Katayama: Conceived the study, included patients, performed laboratorial and formal analysis, revised and approved the final manuscript.

Maria Aparecida Azevedo Koike Folgueira: Conceived the study, wrote the original draft, performed formal analysis, revised and approved the final manuscript.

## Declaration of competing interest

Giselly Encinas is an employee of Agilent Technologies. All other authors declare no conflicts of interest.

## Appendix Supplementary materials

Image, application 1

Image, application 2
