# Supplementary material for: Custom target-sequencing in triple-negative and luminal breast cancer from young Brazilian patients
Source: Clinics (Sao Paulo). 2024 Aug 28;79:100479. doi: 10.1016/j.clinsp.2024.100479 (PMC11399600; doi:10.1016/j.clinsp.2024.100479)

CLINICS-D-23-00041_Supplementary Material

**Supplementary Table 1** Germline variants from 28 young adult breast cancer patients.

| **Subtype** | **SYMBOL** | **rs** | **Location** | **Consequence** | **Findley** | **Aminoacid** | **REF** | **ALT** | **STRAND** | **1000g** | **gnomAD** | **Clinvar** |
| --- | --- | --- | --- | --- | --- | --- | --- | --- | --- | --- | --- | --- |
| TNBC | AHNAK | rs11828907 | 11:62288978-62288978 | Missense | ‒ | D4304G | A | G | -1 | 0.0124 | 0.002478 | B |
| TNBC | AHNAK | rs535365338 | 11:62292687-62292687 | Missense | ‒ | E3068K | G | A | -1 | 0.0002 | 2.78E-05 | ‒ |
| TNBC | AHNAK | rs111966997 | 11:62285664-62285664 | Missense | ‒ | P5409S | C | T | -1 | 0.0026 | 0.0004057 | ‒ |
| TNBC | AHNAK | rs11824660 | 11:62294573-62294573 | Missense | ‒ | P2439L | C | T | -1 | 0.0383 | 0.008839 | ‒ |
| TNBC | AHNAK | rs111578362 | 11:62300280-62300280 | Missense | ‒ | K537Q | A | C | -1 | 0.0026 | 0.0004139 | ‒ |
| TNBC | AHNAK | rs115000832 | 11:62296998-62296998 | Missense | ‒ | G1631S | G | A | -1 | 0.0088 | 0.001885 | B |
| TNBC | APC | rs754783550 | 5:112177107-112177107 | Missense | ‒ | D1939V | A | T | 1 | ‒ | 4.00E-06 | VUS |
| TNBC | APC | rs111866410 | 5:112175651-112175651 | Missense | ‒ | K1454E | A | G | 1 | 0.0008 | 0.0005971 | VUS |
| TNBC | ARID1A | rs61756316 | 1:27102188-27102188 | Missense | ‒ | N1705S | A | G | 1 | 0.0258 | 0.006984 | LB |
| TNBC | ARID1A | rs61756316 | 1:27102188-27102188 | Missense | ‒ | N1705S | A | G | 1 | 0.0258 | 0.006984 | LB |
| TNBC | ARID1A | rs61756316 | 1:27102188-27102188 | Missense | ‒ | N1705S | A | G | 1 | 0.0258 | 0.006984 | LB |
| TNBC | ATAD2B | rs201642875 | 2:23980848-23980848 | Missense | ‒ | T1173M | C | T | -1 | 0.0012 | 0.002551 | ‒ |
| TNBC | ATAD2B | rs10210982 | 2:24118705-24118705 | Missense | ‒ | S118P | T | C | -1 | 0.0162 | 0.003161 | ‒ |
| TNBC | ATM | rs2227922 | 11:108123551-108123551 | Missense | ‒ | P604S | C | T | 1 | 0.0026 | 0.003193 | B |
| TNBC | ATM | rs4986761 | 11:108124761-108124761 | Missense | ‒ | S707P | T | C | 1 | 0.0044 | 0.007958 | B |
| TNBC | ATM | rs2227922 | 11:108123551-108123551 | Missense | ‒ | P604S | C | T | 1 | 0.0026 | 0.003193 | B |
| TNBC | ATM | rs587781352 | 11:108137923-108137923 | Missense | ‒ | D831G | A | G | 1 | ‒ | 3.98E-06 | VUS |
| TNBC | ATM | rs3218695 | 11:108129778-108129778 | Missense | ‒ | D814E | C | A | 1 | 0.0104 | 0.001912 | B |
| TNBC | ATM | rs3218673 | 11:108138045-108138045 | Missense | ‒ | P872S | C | T | 1 | 0.0164 | 0.003532 | B |
| TNBC | ATM | rs4988111 | 11:108198391-108198391 | Missense | ‒ | L2332P | T | C | 1 | 0.0072 | 0.001621 | LB |
| TNBC | ATR | rs28910273 | 3:142188337-142188337 | Missense | ‒ | Y2132D | T | G | -1 | 0.002 | 0.003153 | LB |
| TNBC | ATR | rs1402949922 | 3:142279186-142279186 | Missense | ‒ | I487T | T | C | -1 | ‒ | 3.98E-06 | ‒ |
| TNBC | ATR | rs33972295 | 3:142178118-142178118 | Missense | ‒ | P2434A | C | G | -1 | 0.0126 | 0.003215 | B |
| TNBC | ATXN1 | rs34265178 | 6:16327143-16327143 | Missense | ‒ | G467S | G | A | -1 | 0.0018 | 0.0005509 | ‒ |
| TNBC | ATXN1 | rs139918586 | 6:16326864-16326864 | Missense | ‒ | V560M | G | A | -1 | - | 0.0001752 | VUS |
| TNBC | BAP1 | rs79014342 | 3:52437258-52437258 | Missense | ‒ | S596G | A | G | -1 | 0.027 | 0.005954 | B |
| TNBC | BRCA1 | . | 17:41256941-41256941 | Missense | LOF | L82R | T | G | -1 | ‒ | NA | LP |
| TNBC | BRCA2 | rs80358732 | 13:32913588-32913588 | Missense | ‒ | D1699G | A | G | 1 | 0.0002 | 2.19E-05 | VUS |
| TNBC | BRCA2 | rs11571769 | 13:32953550-32953550 | Missense | ‒ | A2951T | G | A | 1 | 0.01 | 0.00955 | B |
| TNBC | BRCA2 | rs28897701 | 13:32893369-32893369 | Missense | ‒ | A75P | G | C | 1 | 0.0002 | 0.0002586 | B |
| TNBC | BRCA2 | rs11571769 | 13:32953550-32953550 | Missense | ‒ | A2951T | G | A | 1 | 0.01 | 0.00955 | B |
| TNBC | CSMD3 | rs368012163 | 8:113599372-113599372 | Missense | ‒ | S1270R | A | C | -1 | ‒ | 5.58E-05 | ‒ |
| TNBC | CSPP1 | rs756423026 | 8:68007858-68007858 | Missense | ‒ | R281W | C | T | 1 | ‒ | 0.0001166 | VUS |
| TNBC | DICER1 | . | 14:95570124-95570124 | Missense | ‒ | N1203K | T | G | -1 | ‒ | NA | ‒ |
| TNBC | ESR1 | rs200075329 | 6:152129399-152129399 | Missense | ‒ | S118P | T | C | 1 | 0.0016 | 0.004392 | B |
| TNBC | FAT2 | rs34493925 | 5:150924527-150924527 | Missense | ‒ | G2054A | G | C | -1 | 0.0098 | 0.002767 | B |
| TNBC | FAT2 | rs76037457 | 5:150925143-150925143 | Missense | ‒ | V1849I | G | A | -1 | 0.0082 | 0.002513 | B |
| TNBC | FAT2 | rs61743237 | 5:150930192-150930192 | Missense | ‒ | D1513N | G | A | -1 | 0.0082 | 0.002478 | B |
| TNBC | FAT2 | rs61743236 | 5:150930360-150930360 | Missense | ‒ | V1457I | G | A | -1 | 0.0074 | 0.002151 | B |
| TNBC | FAT2 | rs61743254 | 5:150932879-150932879 | Missense | ‒ | W1339R | T | C | -1 | 0.0074 | 0.002149 | B |
| TNBC | FAT2 | rs35640822 | 5:150933984-150933984 | Missense | ‒ | L1295P | T | C | -1 | 0.0074 | 0.002145 | B |
| TNBC | FAT2 | rs116310150 | 5:150946029-150946029 | Missense | ‒ | P822S | C | T | -1 | 0.0038 | 0.001248 | ‒ |
| TNBC | FAT2 | rs200068080 | 5:150897269-150897269 | Missense | ‒ | R3792Q | G | A | -1 | ‒ | 0.0001909 | ‒ |
| TNBC | FAT4 | rs1163197653 | 4:126336783-126336783 | Missense | ‒ | T2222S | C | G | 1 | ‒ | 3.98E-06 | ‒ |
| TNBC | LYST | rs74641549 | 1:235963637-235963637 | Missense | ‒ | D1330A | A | C | -1 | 0.0443 | 0.009912 | B |
| TNBC | LYST | rs147221131 | 1:235827874-235827874 | Missense | ‒ | V3696I | G | A | -1 | 0.0002 | 0.0006055 | LB |
| TNBC | LYST | rs77091385 | 1:235972432-235972432 | Missense | ‒ | Q562H | G | C | -1 | 0.0012 | 0.003787 | LB |
| TNBC | MAP3K1 | rs916555277 | 5:56178599-56178599 | Missense | ‒ | I1191N | T | A | 1 | ‒ | NA | ‒ |
| TNBC | MED23 | rs139398917 | 6:131910690-131910690 | Missense | ‒ | S1285T | G | C | -1 | 0.001 | 0.0001154 | ‒ |
| TNBC | MET | rs35225896 | 7:116340086-116340086 | Missense | ‒ | I316M | A | G | 1 | 0.0054 | 0.001627 | B |
| TNBC | MET | rs757883355 | 7:116339379-116339379 | Missense | ‒ | V81F | G | T | 1 | ‒ | 8.03E-06 | VUS |
| TNBC | NCOA3 | . | 20:46252708-46252708 | Missense | ‒ | E46G | A | G | 1 | ‒ | NA | ‒ |
| TNBC | PCDH10 | rs1176501665 | 4:134072200-134072200 | Missense | ‒ | G302V | G | T | 1 | ‒ | NA | ‒ |
| TNBC | PRKD1 | rs45471692 | 14:30396622-30396622 | Missense | ‒ | P33S | C | T | -1 | ‒ | 0 | B |
| TNBC | PTPN13 | rs61750815 | 4:87666225-87666225 | Missense | ‒ | H865R | A | G | 1 | 0.0002 | 0.002849 | LB |
| TNBC | SETD2 | rs9311404 | 3:47163824-47163824 | Missense | ‒ | V768L | G | C | -1 | 0.0026 | 0.0005611 | B |
| TNBC | SPEN | rs115323964 | 1:16255205-16255205 | Missense | ‒ | R824C | C | T | 1 | 0.0054 | 0.001174 | ‒ |
| TNBC | SPEN | rs77870948 | 1:16260513-16260513 | Missense | ‒ | N2593S | A | G | 1 | 0.0395 | 0.008844 | ‒ |
| TNBC | SPEN | rs146173073 | 1:16247386-16247386 | Missense | ‒ | G553S | G | A | 1 | 0.0024 | 0.0007093 | B |
| TNBC | TNC | rs142544129 | 9:117819530-117819530 | Missense | ‒ | H1494R | A | G | -1 | ‒ | 5.57E-05 | ‒ |
| TNBC | TNC | rs75198247 | 9:117786265-117786265 | Missense | ‒ | N2161S | A | G | -1 | 0.0046 | 0.001359 | B |
| LUM | AHNAK | rs115693058 | 11:62297175-62297175 | Missense | ‒ | M1572V | A | G | -1 | 0.0014 | 0.001948 | B |
| LUM | AHNAK | . | 11:62285402-62285402 | Missense | ‒ | G5496E | G | A | -1 | 0.0016 | 0.002671 | B |
| LUM | AHNAK | rs141151380 | 11:62285402-62285402 | Missense | ‒ | G5496E | G | A | -1 | 0.0016 | 0.002671 | B |
| LUM | AHNAK | rs116243978 | 11:62286165-62286165 | Missense | ‒ | G5242R | G | C | -1 | 0.0046 | 0.007204 | B |
| LUM | AHNAK | rs201059372 | 11:62287616-62287616 | Missense | ‒ | K4758R | A | G | -1 | - | 0.0002307 | ‒ |
| LUM | AHNAK | rs11231129 | 11:62292321-62292321 | Missense | ‒ | V3190I | G | A | -1 | 0.001 | 0.0002545 | ‒ |
| LUM | AHNAK | rs7112976 | 11:62295180-62295180 | Missense | ‒ | P2237T | C | A | -1 | 0.001 | 0.0002664 | ‒ |
| LUM | AHNAK | rs11828907 | 11:62288978-62288978 | Missense | ‒ | D4304G | A | G | -1 | 0.0124 | 0.002478 | B |
| LUM | AHNAK | rs11824660 | 11:62294573-62294573 | Missense | ‒ | P2439L | C | T | -1 | 0.0383 | 0.008839 | ‒ |
| LUM | AHNAK | rs11824660 | 11:62294573-62294573 | Missense | ‒ | P2439L | C | T | -1 | 0.0383 | 0.008839 | ‒ |
| LUM | AHNAK | rs149928120 | 11:62289818-62289818 | Missense | ‒ | P4024R | C | G | -1 | 0.0006 | 8.75E-05 | B |
| LUM | AHNAK | rs11824660 | 11:62294573-62294573 | Missense | ‒ | P2439L | C | T | -1 | 0.0383 | 0.008839 | ‒ |
| LUM | AHNAK | rs115937732 | 11:62291129-62291129 | Missense | ‒ | N3587S | A | G | -1 | 0.0122 | 0.002231 | B |
| LUM | AHNAK | rs148347501 | 11:62291585-62291585 | Missense | ‒ | I3435T | T | C | -1 | 0.002 | 0.0002587 | LB |
| LUM | AHNAK | rs148663483 | 11:62292980-62292980 | Missense | ‒ | L2970W | T | G | -1 | 0.0122 | 0.002175 | B |
| LUM | APC | rs944674770 | 5:112176648-112176648 | Missense | ‒ | R1786T | G | C | 1 | ‒ | 3.99E-06 | VUS |
| LUM | APC | rs1370102446 | 5:112174750-112174753 | Inframe_deletion | ‒ | E1154- | GAA | - | 1 | ‒ | 0.001008 | VUS |
| LUM | ATAD2B | rs10210982 | 2:24118705-24118705 | Missense | ‒ | S118P | T | C | -1 | 0.0162 | 0.003161 | ‒ |
| LUM | ATM | rs369903995 | 11:108160494-108160494 | Missense | ‒ | V1468I | G | A | 1 | 0.0002 | 8.37E-05 | VUS |
| LUM | ATM | rs3092857 | 11:108143299-108143299 | Missense | ‒ | M1040V | A | G | 1 | 0.0142 | 0.003072 | B |
| LUM | ATM | rs3092857 | 11:108143299-108143299 | Missense | ‒ | M1040V | A | G | 1 | 0.0142 | 0.003072 | B |
| LUM | ATM | rs56009889 | 11:108196896-108196896 | Missense | ‒ | L2307F | C | T | 1 | ‒ | 0.001412 | B |
| LUM | ATR | rs33972295 | 3:142178118-142178118 | Missense | ‒ | P2434A | C | G | -1 | 0.0126 | 0.003215 | B |
| LUM | BAP1 | rs35448940 | 3:52437206-52437206 | Missense | ‒ | T613M | C | T | -1 | 0.0042 | 0.0009228 | B |
| LUM | BAP1 | rs79014342 | 3:52437258-52437258 | Missense | ‒ | S596G | A | G | -1 | 0.027 | 0.005954 | B |
| LUM | BRCA1 | rs56082113 | 17:41245090-41245090 | Missense | NA | K820E | A | G | -1 | 0.0104 | 0.002401 | B |
| LUM | BRCA1 | rs397509173 | 17:41256137-41256137 | Splice_donor | NA | --- |  |  | -1 | ‒ | NA | P |
| LUM | BRCA1 | rs1217805587 | 17:41209079-41209079 | Frameshift | NA | Q1777X | - | C | -1 | ‒ | 0.0001829 | P |
| LUM | BRCA2 | rs11571769 | 13:32953550-32953550 | Missense | ‒ | A2951T | G | A | 1 | 0.01 | 0.00955 | B |
| LUM | BRCA2 | rs56248502 | 13:32912582-32912582 | Missense | ‒ | I1364L | A | C | 1 | 0.0044 | 0.001243 | B |
| LUM | BRCA2 | rs144862123 | 13:32911418-32911418 | Missense | ‒ | S976T | T | A | 1 | 0.0024 | 0.0004819 | B |
| LUM | BRCA2 | rs11571656 | 13:32911419-32911419 | Missense | ‒ | S976F | C | T | 1 | 0.0024 | 0.0004811 | B |
| LUM | BRCA2 | rs55969723 | 13:32912679-32912679 | Missense | ‒ | Q1396R | A | G | 1 | 0.002 | 0.000414 | B |
| LUM | BRCA2 | rs11571769 | 13:32953550-32953550 | Missense | ‒ | A2951T | G | A | 1 | 0.01 | 0.00955 | B |
| LUM | BRCA2 | rs80358408 | 13:32906738-32906738 | Missense | ‒ | P375S | C | T | 1 | 0.001 | 2.80E-05 | B |
| LUM | BRCA2 | rs80358515 | 13:32893396-32893396 | Stop_gained | ‒ | Q84* | C | T | 1 | ‒ | NA | P |
| LUM | CACNA1E | rs202202209 | 1:181701810-181701810 | Missense | ‒ | R863Q | G | A | 1 | 0.003 | 0.001201 | B |
| LUM | CDH1 | rs121964872 | 16:68867265-68867265 | Missense | ‒ | S838G | A | G | 1 | ‒ | 4.37E-05 | LB |
| LUM | CDH1 | rs763203357 | 16:68863612-68863612 | Missense | ‒ | R784H | G | A | 1 | ‒ | 3.98E-06 | VUS |
| LUM | CSMD3 | rs150862620 | 8:113668398-113668398 | Missense | ‒ | K997E | A | G | -1 | 0.0012 | 0.0006726 | ‒ |
| LUM | CSMD3 | rs80277352 | 8:113418822-113418822 | Missense | ‒ | I1914V | A | G | -1 | 0.0116 | 0.003027 | B |
| LUM | CSMD3 | rs76916857 | 8:113504760-113504760 | Missense | ‒ | D1746N | G | A | -1 | 0.0046 | 0.001683 | B |
| LUM | FAT2 | rs57774012 | 5:150891768-150891768 | Missense | ‒ | T3955P | A | C | -1 | 0.0166 | 0.003552 | B |
| LUM | FAT2 | rs60050170 | 5:150891806-150891806 | Missense | ‒ | T3942S | C | G | -1 | 0.0166 | 0.003561 | B |
| LUM | FAT2 | rs116401802 | 5:150925883-150925883 | Missense | ‒ | F1602S | T | C | -1 | 0.003 | 0.0008404 | B |
| LUM | FAT2 | rs114551196 | 5:150934209-150934209 | Missense | ‒ | P1220H | C | A | -1 | 0.0036 | 0.0006795 | ‒ |
| LUM | FAT4 | . | 4:126373873-126373873 | Missense | ‒ | I3901T | T | C | 1 | ‒ | NA | ‒ |
| LUM | FAT4 | rs145639192 | 4:126320038-126320038 | Missense | ‒ | I1759V | A | G | 1 | 0.0004 | 0.0008059 | VUS |
| LUM | FAT4 | rs114637892 | 4:126373594-126373594 | Missense | ‒ | H3808R | A | G | 1 | 0.0082 | 0.002054 | B |
| LUM | FAT4 | rs202188213 | 4:126240990-126240990 | Missense | ‒ | V1142M | G | A | 1 | ‒ | 0.0003328 | VUS |
| LUM | FAT4 | rs115822434 | 4:126400922-126400922 | Missense | ‒ | T4167I | C | T | 1 | 0.004 | 0.002574 | B |
| LUM | FAT4 | rs28515675 | 4:126389832-126389832 | Missense | ‒ | R4022Q | G | A | 1 | 0.0132 | 0.001953 | B |
| LUM | HUWE1 | rs142176360 | X:53586416-53586416 | Missense | ‒ | R2605H | G | A | -1 | ‒ | 5.47E-06 | ‒ |
| LUM | LYST | rs143223086 | 1:235969077-235969077 | Missense | ‒ | S1120I | G | T | -1 | 0.003 | 0.000773 | B |
| LUM | MAP2K4 | rs940014479 | 17:11924303-11924303 | Missense | ‒ | V34F | G | T | 1 | ‒ | 0.000014 | ‒ |
| LUM | MET | rs370529693 | 7:116415124-116415124 | Missense | ‒ | P1091L | C | T | 1 | 0.0002 | 0.0001243 | LB |
| LUM | MTOR | rs141877007 | 1:11303235-11303235 | Missense | ‒ | V450I | G | A | -1 | ‒ | 1.19E-05 | VUS |
| LUM | NCOA3 | rs551807396 | 20:46277797-46277797 | Missense | ‒ | T1199A | A | G | 1 | 0.0002 | 2.39E-05 | ‒ |
| LUM | NCOA3 | rs2230781 | 20:46264805-46264805 | Missense | ‒ | P559S | C | T | 1 | 0.0192 | 0.004208 | B |
| LUM | NOTCH1 | rs111309246 | 9:139391200-139391200 | Missense | ‒ | A2331T | G | A | -1 | 0.0076 | 0.001335 | B |
| LUM | NOTCH1 | rs188270459 | 9:139390543-139390543 | Missense | ‒ | I2550V | A | G | -1 | 0.0004 | 0.0002464 | LB |
| LUM | NOTCH1 | rs80340744 | 9:139401302-139401302 | Missense | ‒ | P1256L | C | T | -1 | 0.0072 | 0.001512 | B |
| LUM | NOTCH1 | rs150737112 | 9:139417343-139417343 | Missense | ‒ | R234H | G | A | -1 | 0.0004 | 0.0005159 | VUS |
| LUM | POLD1 | . | 19:50902196-50902196 | Missense | ‒ | R30W | C | T | 1 | 0.0038 | 0.008208 | B |
| LUM | POLD1 | rs3218772 | 19:50902196-50902196 | Missense | ‒ | R30W | C | T | 1 | 0.0038 | 0.008208 | B |
| LUM | POLD1 | rs914238978 | 19:50905485-50905485 | Missense | ‒ | G205S | G | A | 1 | ‒ | 1.31E-05 | VUS |
| LUM | POLD1 | rs914238978 | 19:50905485-50905485 | Missense | ‒ | G205S | G | A | 1 | ‒ | 1.31E-05 | VUS |
| LUM | POLD1 | rs1726803 | 19:50905310-50905310 | Missense | ‒ | S173N | G | A | 1 | 0.0316 | 0.007704 | B |
| LUM | PTPN13 | rs34226837 | 4:87735618-87735618 | Missense | ‒ | I2463V | A | G | 1 | 0.0188 | 0.003839 | ‒ |
| LUM | PTPN13 | rs372924378 | 4:87610176-87610176 | Missense | ‒ | H127N | C | A | 1 | ‒ | 5.63E-05 | ‒ |
| LUM | PTPN13 | rs114206680 | 4:87684234-87684234 | Missense | ‒ | K1303R | A | G | 1 | 0.0022 | 0.0005866 | ‒ |
| LUM | PTPN13 | rs34226837 | 4:87735618-87735618 | Missense | ‒ | I2463V | A | G | 1 | 0.0188 | 0.003839 | ‒ |
| LUM | RAD9A | rs568076402 | 11:67164800-67164803 | Inframe_deletion | ‒ | E342- | GAG | - | 1 | ‒ | 0.0006759 | ‒ |
| LUM | SETD2 | rs780288575 | 3:47162089-47162089 | Missense | ‒ | G1346V | G | T | -1 | ‒ | 3.98E-06 | ‒ |
| LUM | SPEN | rs564799201 | 1:16262685-16262685 | Missense | ‒ | P3317L | C | T | 1 | ‒ | 4.19E-06 | ‒ |
| LUM | SPEN | rs61749275 | 1:16258756-16258756 | Missense | ‒ | D2007E | T | A | 1 | 0.0018 | 0.004591 | ‒ |
| LUM | TNC | rs144032672 | 9:117849382-117849382 | Missense | ‒ | G210S | G | A | -1 | 0.0012 | 0.001297 | LB |

ID, Sample ID; Findley, BRCA1 variant functional classification according to Findley et. al. (2018); Clinvar, Classification of variants according to functional studies; B, Benign; VUS, Variant of Uncertain Significance; LB, Likely-Benign; LP, Likely-Pathogenic; P, Pathogenic.

**Supplementary Figure 1** **Oncoplot of the germline variants detected in the 28 young adult breast cancer patients.** Each column is a patient, and each line represents a gene. Alterations: classification of variants according to ACMG (exported from Clinvar), B, Benign; LB, Likely-Benign; NA, Variant was Not reported/investigated; VUS, Variant of Uncertain Significance; LP, Likely-Pathogenic; P, Pathogenic; FH, Family History of at least one relative (until third-degree relatives) with a diagnosis of breast, ovary, pancreas, or prostate cancer.


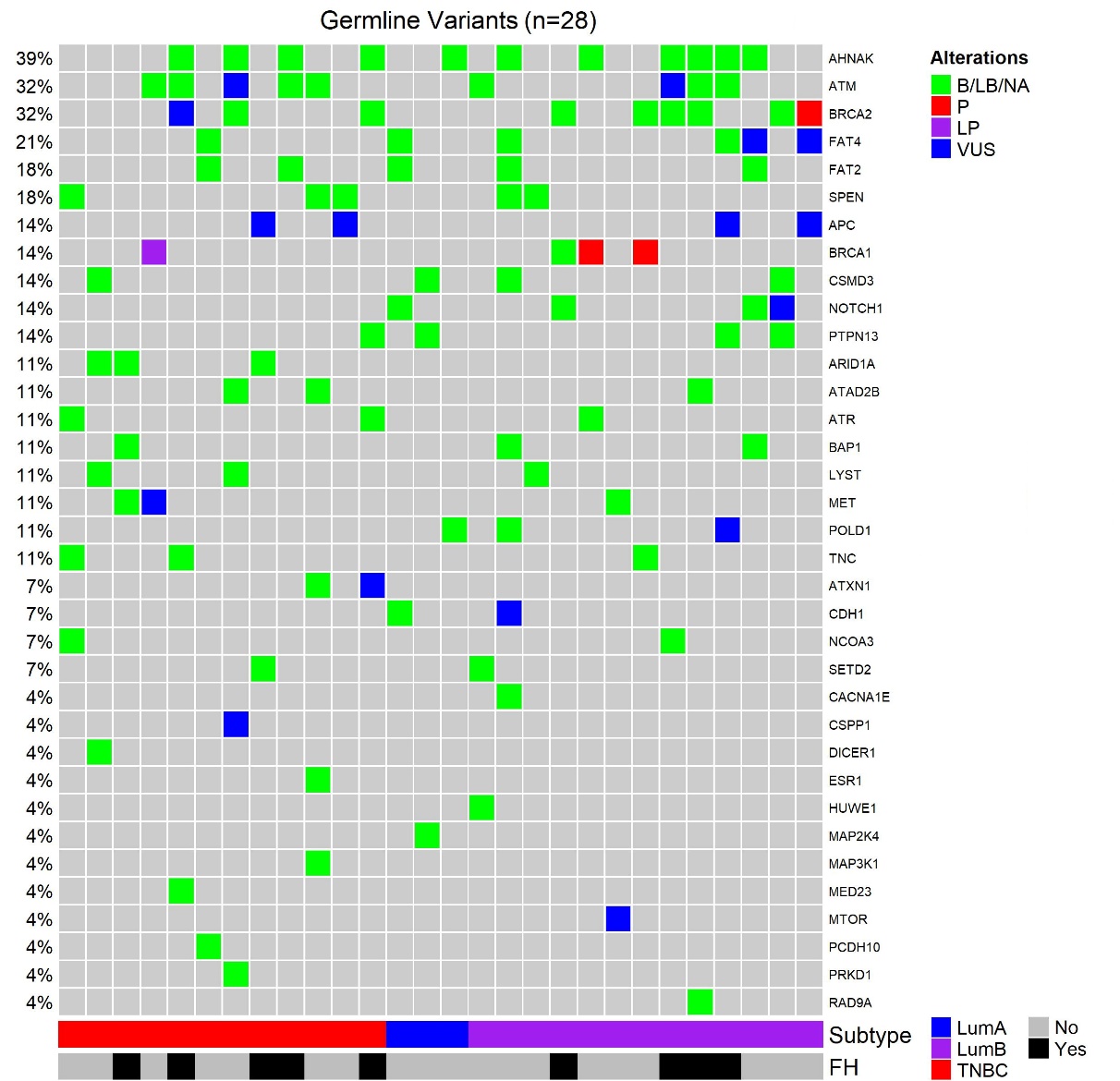

Supplement: Supplementary file 1 [file mmc1.docx]
