# Supplementary material for: Custom target-sequencing in triple-negative and luminal breast cancer from young Brazilian patients
Source: Clinics (Sao Paulo). 2024 Aug 28;79:100479. doi: 10.1016/j.clinsp.2024.100479 (PMC11399600; doi:10.1016/j.clinsp.2024.100479)
Supplement: Supplementary file 2 [file mmc2.docx]

**Supplementary methods**

**Custom target-sequencing in triple-negative and luminal breast cancer from young Brazilian patients**

**Pedro Adolpho de Menezes Pacheco Serio^1^, Daniela Marques Saccaro^1^, Ana Carolina Ribeiro Chaves de Gouvêa^1^, Giselly Encinas^2^, Simone Maistro^1^, Gláucia Fernanda de Lima Pereira^1^,** **Vinícius Marques Rocha^1^, Larissa Dias de Souza^1^, Viviane Jennifer da Silva^1^, Maria Lucia Hirata Katayama^1^, Maria Aparecida Azevedo Koike Folgueira^1^**

**Sample collection and extraction**

Patients who agreed to participate in the study had their respective blood samples collected and their respective tumor samples embedded in paraffin (Formalin-Fixed Paraffin-Embedded - FFPE) accessed through the pathology department of ICESP.

**DNA extraction**

For each sample, a slide stained with HE was analyzed by the pathologist and the region of interest (with ≥30% of tumor cells) was demarcated. With the aid of a Microtome (Zeiss), 5 cuts of 20 µm were obtained. Using the previously analyzed HE slide as a guide, the region of interest was scraped and stored in 1.5 ml tubes. DNA was extracted using QIAamp® DNA FFPE Tissue (Qiagen - 56404), following manufacturer protocol. For blood samples, DNA was extracted using the QIAamp® DNA Mini Kit (Qiagen – 51306), following manufacturer protocol.

**DNA Library Construction**

Paired blood and FFPE samples that were successfully extracted and at a minimum concentration of 10 ng/uL were processed as described in the SureSelectXT HS Target Enrichment System for Illumina Multiplexed Sequencing Platforms protocol (Agilent).

Finally, the libraries were combined to reach the same equimolarity (10 nM) in 1x Low TE buffer, and then sequenced on the NextSeq device (NextSeq 500/550 Mid Output Kit v2.5, 150 cycles; Illumina).

**Quality Control (QC) and data processing**

**I - Quality control**

Clusterization density and Q30 scores were checked during and after the sequencing process, through the interface of the BaseSpace tool (Illumina), directly linked to the sequencing equipment, where a density between 170 and 220 K/mm² (NextSeq 500/550 Mid Output Kit v2.5, 150 cycles) and a Q30 in at least 80% of the total reads of each sample was considered as good quality.

**III - Quality control after alignment and variant**

As a quality control, we chose a minimum value of 20 total reads and a ratio of total reads/altered reads of at least 10%, per variant. As the variability of coverage and quality of target regions can vary greatly between samples and regions in target sequencing, all detected variants were checked for quality of coverage according to the patient, gene and exon they were located. To verify the global quality of probes and samples, we checked the coverage of the exons of each gene in the panel, per patient, calculating the median coverage between probes from a patient or from a gene in all patients, thus extracting the estimate of global quality of probes and samples.

We excluded variants mapped in low-complexity regions and/or variants with low mapping quality. In addition, 2 genes were excluded from further analysis, these being HERC2 and PARP4, as they presented low coverage in all analyzed samples. All non-rare variants (polymorphisms) with an allele frequency (GnomAD) equal to or greater than 1% were filtered out.

IV – Variant effect prediction annotation

The following variant effect prediction algorithms were used: FATHMM (1), MutationAssesor (2), MutationTaster (3), PROVEAN (4), Polyphen2 HDIV, Polyphen2 HVAR (5), SIFT and SIFT4G (6). We also used algorithms focused on the integration of results from multiple variant effect prediction tools, such as: REVEL (7), METALR and METASVM (8) and MCAP (9).

**IV - Copy Number Variation (CNV)**

Somatic CNVs were detected using SureCall (Agilent) software, where tumor samples were used as investigational samples, and their respective paired blood samples were used as reference samples. For the calculation of CNVs, the program considers the coverage per base of each range of target regions of the panel genes (in this case, their exons), computing and normalizing the coverage values ​​between the pairs of samples. As an output, we obtain the gene and the coordinate of the region where it is amplification or deletion, this effect being represented by the value of the ratio between paired samples, in addition, the size of the region that represents the CNV is also computed.

For calling CNVs, we used most of the software's base filtering and quality control settings. Since the coverage of some blood samples was higher than in tumor samples, we used a strict filter for CNV detection, to ensure as few false-positives as possible. Only variants with a confidence score (probability of being a true-positive finding) of at least 0.97 (97%) and only regions with at least 20 reads of coverage were accepted, we also corrected the detection to consider the coverage fold-changes between blood and tumor paired samples.

References

1. Shihab HA, Gough J, Cooper DN, Stenson PD, Barker GLA, Edwards KJ, et al. Predicting the functional, molecular, and phenotypic consequences of amino acid substitutions using hidden Markov models. Hum Mutat. 2013 Jan;34(1):57–65.

2. Reva B, Antipin Y, Sander C. Predicting the functional impact of protein mutations: application to cancer genomics. Nucleic Acids Res. 2011 Sep 1;39(17):e118.

3. Steinhaus R, Proft S, Schuelke M, Cooper DN, Schwarz JM, Seelow D. MutationTaster2021. Nucleic Acids Res. 2021 Jul 2;49(W1):W446–51.

4. Choi Y, Sims GE, Murphy S, Miller JR, Chan AP. Predicting the functional effect of amino acid substitutions and indels. PLoS ONE. 2012 Oct 8;7(10):e46688.

5. Adzhubei IA, Schmidt S, Peshkin L, Ramensky VE, Gerasimova A, Bork P, et al. A method and server for predicting damaging missense mutations. Nat Methods. 2010 Apr;7(4):248–9.

6. Vaser R, Adusumalli S, Leng SN, Sikic M, Ng PC. SIFT missense predictions for genomes. Nat Protoc. 2016 Jan;11(1):1–9.

7. Ioannidis NM, Rothstein JH, Pejaver V, Middha S, McDonnell SK, Baheti S, et al. REVEL: an ensemble method for predicting the pathogenicity of rare missense variants. Am J Hum Genet. 2016 Oct 6;99(4):877–85.

8. Dong C, Wei P, Jian X, Gibbs R, Boerwinkle E, Wang K, et al. Comparison and integration of deleteriousness prediction methods for nonsynonymous SNVs in whole exome sequencing studies. Hum Mol Genet. 2015 Apr 15;24(8):2125–37.

9. Jagadeesh KA, Wenger AM, Berger MJ, Guturu H, Stenson PD, Cooper DN, et al. M-CAP eliminates a majority of variants of uncertain significance in clinical exomes at high sensitivity. Nat Genet. 2016 Dec;48(12):1581–6.
